# Investigation of deaths from ill-defined causes and probable scarlet fever outbreak among schoolchildren, São João del-Rei, 2023

**DOI:** 10.1590/S2237-96222026v35e20240407.en

**Published:** 2025-10-16

**Authors:** Mariana Sanches de Mello, Andressa Rocha Olah, Katiucia Carolina Canaan, Karina Cardoso, Eva Lidia Arcoverde Medeiros, Daniela Caldas Teixeira, Flávia Ribeiro Soares Cruzeiro, Iara Fabiola Batista Rocha, Thais Cristina Nazaré Freitas, Maria Isabella Claudino Haslett, Samara Carolina Rodrigues

**Affiliations:** 1 Ministério da Saúde, Secretaria de Vigilância em Saúde e Ambiente, Brasília, DF, Brazil Ministério da Saúde Secretaria de Vigilância em Saúde e Ambiente Brasília DF Brazil; 2 Secretaria Municipal de Saúde de São João del-Rei, Vigilância Epidemiológica, São João del-Rei, MG, Brazil Secretaria Municipal de Saúde de São João del-Rei Vigilância Epidemiológica São João del-Rei MG Brazil; 3 Secretaria de Estado de Saúde de Minas Gerais, Centro de Informações Estratégicas em Vigilância em Saúde, Belo Horizonte, MG, Brazil Secretaria de Estado de Saúde de Minas Gerais Centro de Informações Estratégicas em Vigilância em Saúde Belo Horizonte MG Brazil

**Keywords:** Epidemiology, Scarlet Fever, Observational Study, Students, Respiratory Tract Diseases, Epidemiología, Escarlatina, Estudio Observacional, Estudiantes, Enfermedades Respiratorias

## Abstract

**Objective:**

To investigate deaths from ill-defined causes and a probable scarlet fever outbreak among schoolchildren in São João del-Rei between September and October 2023.

**Methods:**

This was a descriptive study of children hospitalized with sepsis symptoms and a case series of schoolchildren with pharyngotonsillitis and scarlet fever. Data from medical records and interviews were used to calculate absolute frequencies and measures of central tendency.

**Results:**

Six healthy children (1-10 years old, of White/mixed race skin color) were hospitalized with sepsis symptoms, presenting fever, purpura, bruising, and odynophagia. *Streptococcus sp*. was identified in oropharyngeal swabs in three of the six children, but blood cultures were negative. Three children died, three were discharged, and two attended the same school. Among the schoolchildren, 873 records of absenteeism were identified; of these, 212 met the definition of pharyngotonsillitis and 87 were interviewed. Of the 87,7 also met the criteria for scarlet fever, 47 were female, and 78 were aged 5-13 years. Symptoms included malaise (62/87), throat plaques (49/87), and body rash (26/87). Antibiotics (58/75) and analgesics/antipyretics (32/75) were prescribed for treatment. During renovations at the school, small poorly ventilated adapted containers were used as classrooms. Twelve students did not use individual drinking glasses, and 53/87 did not use masks when symptomatic.

**Conclusions:**

Suspected invasive streptococcal disease deaths were caused by septic shock, without confirmation of the etiological agent. *Streptococcus sp*. may have contributed to the probable scarlet fever outbreak. Recommendations were made to alert the population and professionals about hygiene practices and staying away from school, in addition to training health teams to diagnose and manage re-emerging diseases.

## Resumen

**Objetivo:**

Investigar las muertes por causas mal definidas y un probable brote de escarlatina en escolares de São João del-Rei entre septiembre y octubre de 2023.

**Métodos:**

Estudio descriptivo de niños hospitalizados con síntomas sépticos y una serie de casos de escolares con faringoamigdalitis y escarlatina. Se utilizaron datos de historias clínicas y entrevistas para calcular frecuencias absolutas y medidas de tendencia central.

**Resultados:**

Seis niños sanos (de 1 a 10 años, de piel blanca/morena) fueron hospitalizados con síntomas sépticos, que presentaban fiebre, púrpura, hematomas y odinofagia. Se identificó Streptococcus sp. en hisopados orofaríngeos en tres de los seis niños, pero los hemocultivos fueron negativos. Tres niños fallecieron, tres fueron dados de alta y dos asistían a la misma escuela. Entre los escolares, se identificaron 873 registros de ausentismo; de estos, 212 cumplían con la definición de faringoamigdalitis y 87 fueron entrevistados. De los 87, 7 también cumplían los criterios de escarlatina, 47 eran del sexo femenino y 78 tenían entre 5 y 13 años. Los síntomas incluyeron malestar (62/87), dolor de garganta (49/87) y manchas en el cuerpo (26/87). Se prescribieron antibióticos (58/75) y analgésicos/antipiréticos (32/75) para el tratamiento. Durante las reformas de la escuela, se utilizaron pequeños contenedores mal ventilados adaptados como aulas. Doce estudiantes no usaron vasos individuales y 53/87 no usaron mascarillas cuando presentaron síntomas.

**Conclusiones:**

Las muertes sospechosas de enfermedad invasiva por estreptococos fueron causadas por choque séptico, sin confirmación del agente etiológico. Streptococcus sp. puede haber contribuido al probable brote de escarlatina. Se recomendó alertar a la población y a los profesionales sobre las prácticas de higiene y el aislamiento escolar, además de capacitar a los equipos de salud para diagnosticar y manejar enfermedades reemergentes.

**Ethical aspects**

This research respected ethical principles, having obtained the following approval data:

Research ethics committee: National Research Ethics Committee

Opinion number: 6,799,038

Approval date: 2/5/2024

Certificate of submission for ethical appraisal : 78833224.1.0000.0008

Informed consent record: Waived.

## Introduction

Infectious pharyngotonsillitis is one of the most common causes of fever in children and contributes to school absenteeism, recurrent infections, and increased risk of complications ^1^. It frequently affects children over 5 years of age and is more common in winter and spring ^1^^,^^2^. 

Thirty percent of cases have a bacterial etiology, with *Streptococcus pyogenes* being the most prevalent ^2^^,^^3^^,^^4^^,^^5^^,^^6^^,^^7^. Approximately 10.0% of infected individuals are sensitive to the toxins released, and some virulent strains carry genes that encode superantigens, including pyrogenic exotoxins, the main factor associated with typical scarlet fever rash ^2^^,^^3^^,^^4^^,^^5^^,^^6^^,^^7^. This microorganism can also cause impetigo, rheumatic fever, glomerulonephritis, and invasive diseases such as toxic shock ^2^^,^^3^.

Scarlet fever is characterized by high fever, sore throat, and scarlatiniform rash - a diffuse erythema with 1- to 2-mm micropapules that give the skin a sandpaper-like texture. Individuals often have flushed cheeks and paleness around the mouth, as well as a more pronounced rash along major skin folds, known as Pastia’s sign. The papillae of the tongue may be hypertrophic, giving the appearance of a “strawberry tongue” ^3^^,^^8^. The incubation period ranges from one to ten days, and transmission occurs from the first symptoms to 21 days or 24 hours after the start of antibiotic therapy ^5^^,^^6^. Scarlet fever is transmitted by direct contact with respiratory secretions, through coughing and sneezing, and by sharing objects such as drinking glasses, water bottles, and cutlery among sick and/or asymptomatic individuals ^1^^,^^2^^,^^3^^,^^4^^,^^5^^,^^6^^,^^9^.

Diagnosis is clinical, with no need for tests to begin treatment. However, laboratory tests in symptomatic cases help identify the pathogen ^1^^,^^5^^,^^6^^,^^7^. Detection can be performed by biochemical tests, microbiological cultures and rapid tests, which should preferably be collected before administering antibiotics ^1^^,^^4^^,^^5^^,^^6^^,^^7^. Treatment is based on supportive measures, such as rest, hydration and administration of analgesics, antipyretics and antibiotics ^1^^,^^5^^,^^6^^,^^7^.

Scarlet fever is seasonal and affects school-age children. This disease had practically disappeared by the end of the 20^th^ century ^3^, however, recent outbreaks have occurred in Asia and the United Kingdom, and concomitantly, there has been an increase in cases of invasive infections ^3^^,^^4^^,^^5^. 

In 2022, cases of invasive disease caused by group A streptococci and scarlet fever were reported in European countries, the United States, Uruguay and Argentina, with outcomes with progression to death being identified, related to cases with invasive presentation ^10^^,^^11^. In Brazil, outbreaks of scarlet fever in children were reported in Minas Gerais ^12^ and São Paulo ^13^ in 2023, which drew attention to the re-emergence of the disease in Brazil.

In São João del-Rei, Minas Gerais state, three deaths and three hospitalizations of children, accompanied by rumors of invasive disease caused by *Streptococcus pyogenes*, led to a request for support from the Training Program on Epidemiology Applied to Brazilian National Health System Services (EpiSUS) for epidemiological investigation. This study aimed to investigate deaths with ill-defined causes, find epidemiological links and investigate the probable outbreak of scarlet fever among cases of pharyngotonsillitis in a school in São João del-Rei.

## Methods

Design

This was a descriptive study of children hospitalized with sepsis symptoms and a case series, between September 1 and October 31, 2023 in São João del-Rei, Minas Gerais state. The school where the case series was carried out was located in the same municipality and served more than 900 students in basic education (early childhood, elementary and high school) and technical education.

Setting 

Brazil is the largest country in South America, with a land area of ​​8 million km^2^ and a population of 203,080,756 inhabitants. The territory is divided into 27 Federative Units, i.e. 26 states and the Federal District. At a lower level, the country is subdivided into 5,588 municipalities. One of these is São João del-Rei, located in the southeast of the state of Minas Gerais ^14^.

São João del-Rei is a historic municipality with a population of 90,897 inhabitants and is one of the main tourist destinations in the interior region of Minas Gerais ^14^. It falls into to the state’s Central-South health macro-region and, in order to provide health care to the population, it has 12 primary health care units and 23 family health strategy teams, with approximately 73.0% primary health care coverage. It also has an urgent care center, a public polyclinic and two hospitals.

Participants

This study included children hospitalized in São João del-Rei with sepsis symptoms and students from the school where two of these children studied and who had a record of absenteeism with reports of fever and odynophagia. We used adapted case definitions of invasive streptococcal disease ^8^^,^^9^^,^^10^, suspected and confirmed death from invasive streptococcal disease, pharyngotonsillitis cases, scarlet fever cases and scarlet fever outbreak ^13^

Invasive streptococcal disease case: an individual hospitalized with sepsis symptoms and isolation of *Streptococcus pyogenes* in culture (blood culture, cerebrospinal fluid or deep tissues), between September and October 2023.

Death due to suspected invasive streptococcal disease: hospitalized individual who died before diagnosis of invasive disease or without sufficient data to classify as a probable or confirmed case, between September and October 2023.

Confirmed death due to invasive streptococcal disease: hospitalized individual who died with medical diagnosis of invasive disease and who had *Streptococcus pyogenes* isolated in culture (blood culture, cerebrospinal fluid or deep tissue), between September and October 2023.

Pharyngotonsillitis case: individual who presented fever and odynophagia, with a record of school absenteeism between September and October 2023. 

Scarlet fever case: individual who presented fever, odynophagia and scarlatiniform rash, with a record of school absenteeism between September and October 2023.

Scarlet fever outbreak: occurrence of two or more cases of scarlet fever in a given geographic area, within 21 days after the onset of signs and symptoms of the first identified case.

There was no clinical validation of the cases, but rather we considered the clinical-epidemiological criteria self-reported by the participants. We also considered the definitions of clinical validation, clinical-epidemiological criteria, clinical-laboratory criteria, probable scarlet fever outbreak and confirmed scarlet fever outbreak ^15^ adapted to the context of the investigation:

Clinical validation: medical diagnosis of pharyngotonsillitis/scarlet fever, confirmed in medical records.

Clinical-epidemiological criterion: case that met the definition of pharyngotonsillitis and/or scarlet fever and confirmed contact with hospitalized children and/or those who fell ill at school.

Clinical-laboratory criterion: individual who met the definition of pharyngotonsillitis and/or scarlet fever and had *Streptococcus pyogenes* isolated in culture (oropharyngeal swab, blood culture or deep tissue culture).

Probable scarlet fever outbreak: set of scarlet fever cases that met the definition of a scarlet fever outbreak and presented the clinical-epidemiological criterion, but without laboratory confirmation.

Confirmed scarlet fever outbreak: set of scarlet fever cases that met the definition of a scarlet fever outbreak and at least one case that met the clinical-laboratory criterion with the remaining cases meeting the clinical-epidemiological criterion.

Data source and analysis

Secondary data were collected from medical records and primary data were collected through interviews. Semi-structured instruments containing sociodemographic and clinical-epidemiological variables were used to investigate hospitalized children and for the investigation conducted at the school. For medical record data collection, the instrument was divided into sociodemographic variables (age, sex, municipality of residence and school attended) and clinical-epidemiological variables (main signs and symptoms, diagnostic hypothesis, tests performed and clinical outcome).

The questionnaire used for the interviews with the students was prepared using EpiInfo version 7.0 and divided into blocks: respondent data, student data (date of birth, race/skin color, sex, municipality of residence), clinical-epidemiological data (school shift and grade, individual glass for drinking water at school, signs and symptoms and date of onset, tests performed, correlation of symptom onset to a prior event/situation, use of a mask during the manifestation of symptoms, contact with people with similar symptoms, family member with similar symptoms, child in the same class with similar symptoms, contact with hospitalized children, travel prior to symptoms, student with flu-like symptoms before the symptoms) and seeking health care (over-the-counter medication, number of times sought care, date and place sought care, diagnosis received during care, prescribed medication, hospitalization). When the presence of skin rash was mentioned by the guardian and/or the student, the interviewers requested photographs to prove the report and/or showed them pictures that could indicate the type of rash presented by the individual.

The data collection team was trained beforehand and was comprised of members of the Minas Gerais State Health Department, the São João del-Rei Municipal Health Department, and EpiSUS. Students whose guardians were contacted three times on different days and times, in person and/or by telephone, without success, those whose guardians’ addresses or telephone contacts were not found, and those who refused to participate were considered losses. 

Individuals participated voluntarily, with verbal authorization from their legal guardians, without financial benefits or coercion ^16^. The requirement for informed consent was waived because this was a public health surveillance action. The data obtained ^17^ were analyzed by calculating measures of absolute frequency and central tendency, using SPSS version 22.0.

## Results

During the study period, six previously healthy children were hospitalized in São João del-Rei with sepsis symptoms. Regarding the characterization of the individuals, all were of White or mixed race skin color, aged 1-10 years, had fever and body rashes (petechiae, hematomas and hemorrhagic suffusions). Four of them had odynophagia. *Streptococcus sp*. was identified via oropharyngeal swab culture in three children, although their blood cultures were negative. Three died, three were discharged from hospital, and two attended the same school (Figure 1). The hypothesis was raised that there had been an outbreak of bacterial infectious disease in the school that two of the hospitalized children attended.

**Table 1:**
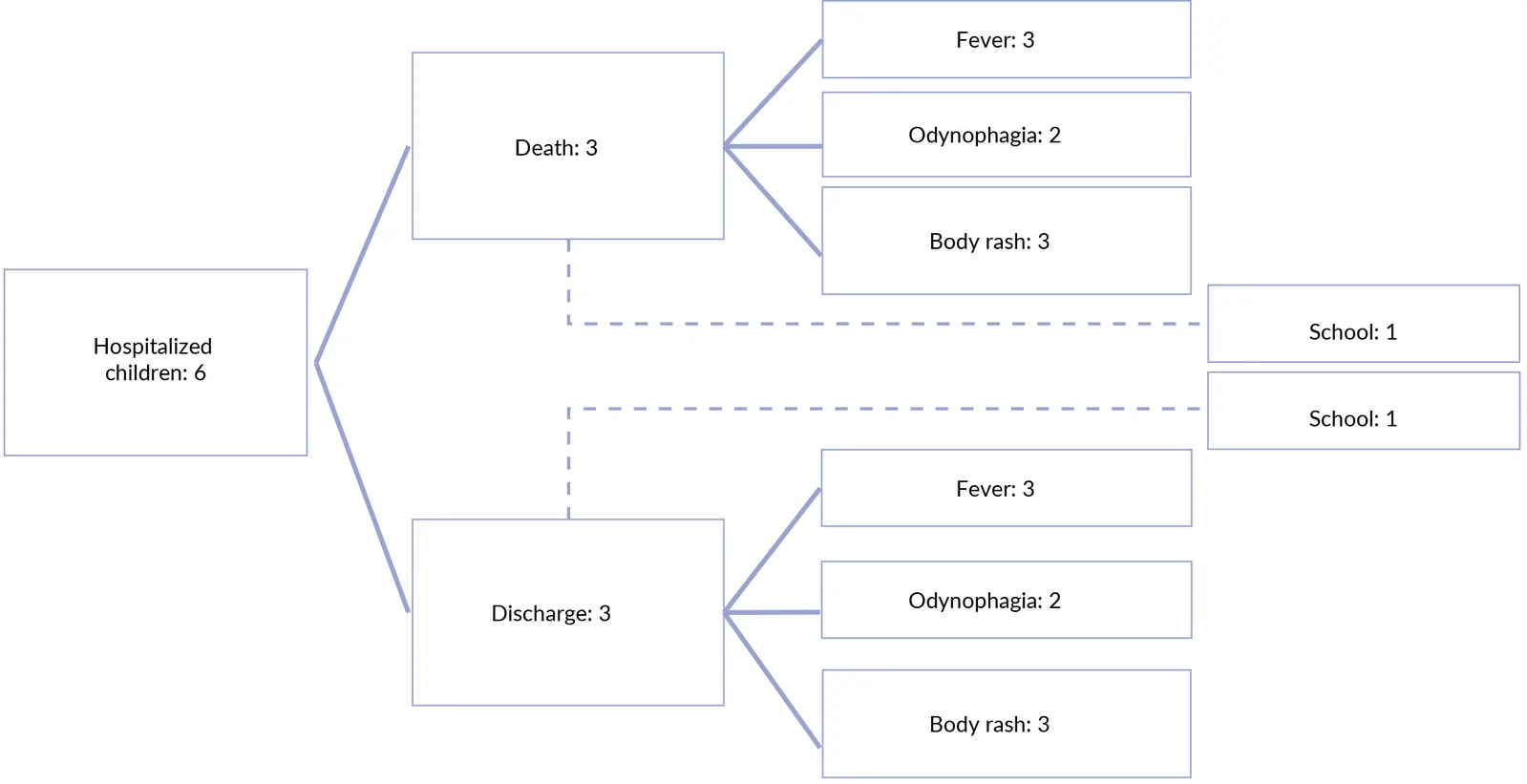
Sociodemographic characteristics of cases of pharyngotonsillitis and scarlet fever. São João del-Rei, September-October 2023 (n=87)

An epidemiological investigation was conducted at this school and 873 records of absenteeism were found during the period. According to the pharyngotonsillitis case definition, 212 (24.3%) students were eligible for the study; however, in 113/212 cases, the contacts made were unsuccessful, 8/212 refused to participate and there was no information on the address or telephone number of 4/212. Of the total number of students eligible for the study (212), 87 were interviewed.

The predominant age group among the cases was 5-13 years (78/87), however, cases also occurred in individuals aged 14-19 years (9/87) (Table 1). All interviewees met the clinical-epidemiological case definition for pharyngotonsillitis, in addition, 7/87 also met the clinical-epidemiological case definition for scarlet fever. Of the total number of cases, there was a higher occurrence among females (47/87), White race/skin color was predominant (60/87), as were students who lived in São João del-Rei itself (65/87) (Table 1).

**Table 1:** Sociodemographic characteristics of cases of pharyngotonsillitis and scarlet fever. São João del-Rei, September-October 2023 (n=87)

Variable	n
Sex	
Female	47
Male	40
Race/skin color	
White	60
Mixed race	24
Asian	2
Black	1
Age group (years)	
5-7	28
08-10	27
11-13	23
14-16	6
17-19	3
Municipality of residence	
São João del-Rei	65
Santa Cruz de Minas	19
Tiradentes	1
Coronel Xavier Chaves	1
No information	1

In August 2023, renovations to the school’s physical structure began and, as of August 6^th^, 20 of the 26 classes were relocated to containers and six remained in conventional classrooms. The classes were divided between morning (A) and afternoon (B) shifts. The two preschool classes (A and B) and both shifts (A and B) of the 18 elementary school classes (1^st^ to 9^th^ grade) began to attend classes in adapted containers. Both shifts of the six high school classes (1^st^ to 3^rd^ grade) remained in conventional classrooms. Although the containers had doors and windows, the spaces were small and poorly ventilated. 

Of the school’s 26 classes/grades, 22 had at least one clinical-epidemiological case of pharyngotonsillitis and/or scarlet fever recorded. Among the 20 classes allocated to containers, 82/87 students presented symptoms and, among those who remained in conventional classrooms, 5/87 were symptomatic. Among the classes and shifts that presented the highest number of cases in the period, elementary 4^th^ grade B stood out, this being one of the grades having lessons in an adapted classroom, attended by two of the children who were hospitalized (Figure 2).

**Figure 2:**
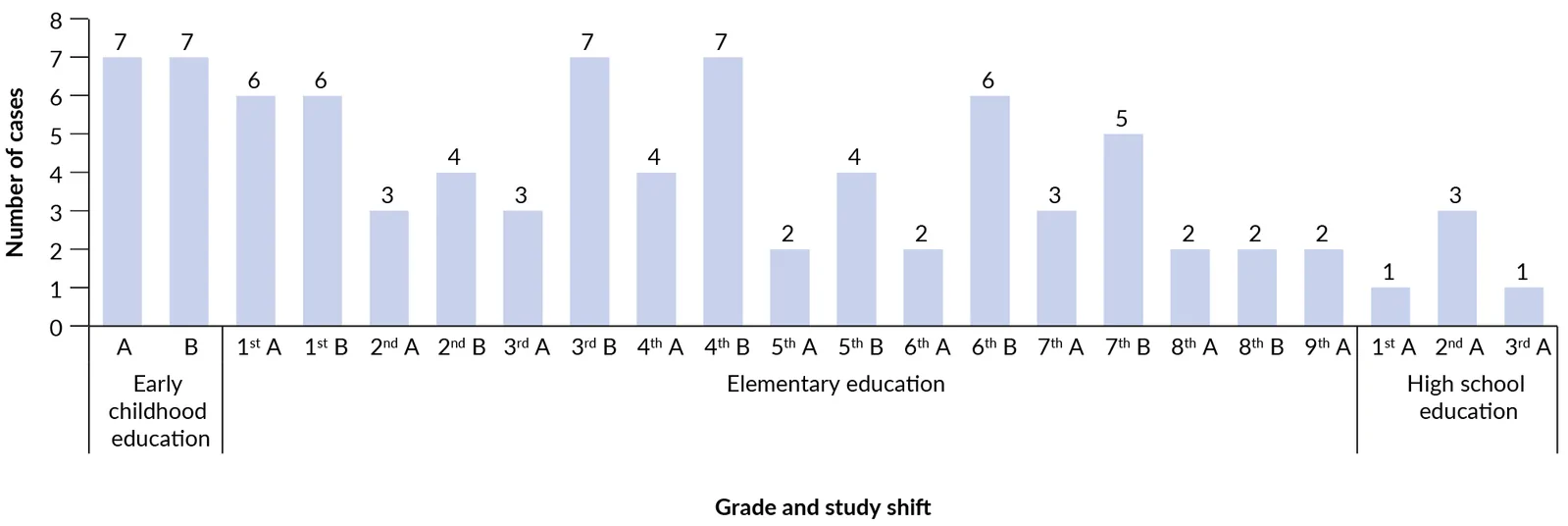
School grade and study shift, morning (A) and afternoon (B), for cases of pharyngotonsillitis and scarlet fever. São João del-Rei, September-October 2023 (n=87)

The 87 clinical and epidemiological cases of pharyngotonsillitis and scarlet fever were distributed temporally in August (1/87), September (38/87) and October (47/87); one of the cases (1/87) was unable to specify the date of sign and symptom onset. It was evident that, from the second half of September, there was an increase in the number of cases, when compared to August (Figure 3).

In addition to the signs and symptoms established in the case definitions (fever and odynophagia), more than half of the 87 clinical-epidemiological cases of pharyngotonsillitis and scarlet fever presented malaise (62/87), prostration (59/87), headache (52/87) and throat plaques (49/87). Body rashes were present in 26/87 cases and the most frequently reported symptoms were hemorrhagic suffusions (1/26), petechiae (16/26), scarlatiniform rash (7/26) and blisters (1/26).

**Figure 3:**
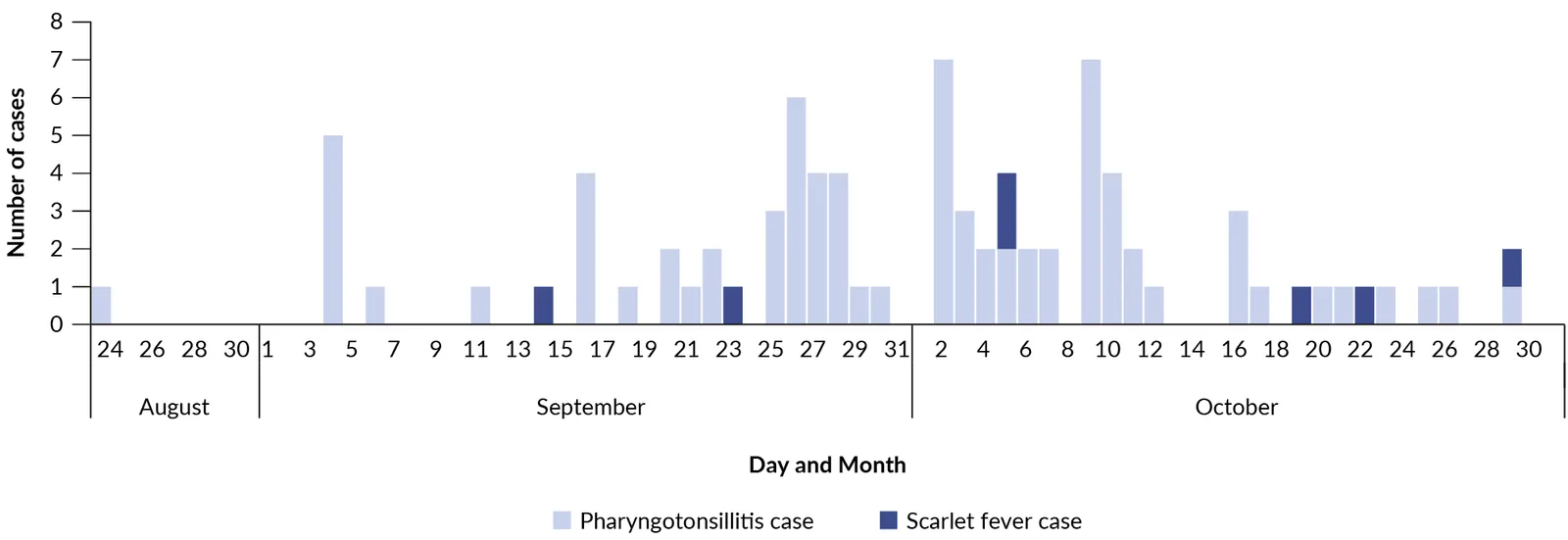
Cases of pharyngotonsillitis and scarlet fever, according to date of symptom onset. São João del-Rei, September-October 2023 (n=86)

When asked about the use of personal items in the school environment, we found that 12/87 students did not use their own drinking glass or water bottle, but rather used the water fountain directly or shared these objects with classmates. Symptom onset in 43/87 cases was related to a prior event/situation, with emphasis on adapted containers being used as classrooms (14/43) and a birthday party (1/43) (Table 2).

Among the interviewees, eight children had traveled before the onset of signs and symptoms, and the main destinations were cities in the states of Minas Gerais, São Paulo, Rio de Janeiro and Goiás. However, there were no reports of contact with symptomatic individuals on that occasion. It was reported that 25/87 children had contact with individuals who presented similar symptoms before developing pharyngotonsillitis or scarlet fever (Table 2). 

**Table 2:** Clinical and epidemiological variables of pharyngotonsillitis and scarlet fever cases. São João del-Rei, September-October 2023 (n=87)

Variables	n
Use of own glass/bottle	
Yes	75
No	12
Symptom onset correlated to prior event/situation	
Yes	43
No	44
Journey 20 days prior to symptoms	
Yes	8
No	79
Prior contact with symptomatic person	
Yes	25
No	56
No information	6
Contact with hospitalized children	
Yes	20
No	60
No information	7
Appearance of symptoms (n=20)	
Before	4
After	16
Symptomatic classmate	
Yes	56
No	10
No information	21
Student with prior influenza-like illness	
Yes	30
No	57
Use of mask following symptoms	
Yes	34
No	53
Attended school with symptoms	
Yes	33
No	54
Medication without prescription prior to care	
Yes	67
No	20
Took analgesics/antipyretics (n=67)	
Yes	62
No	5
Took antiemetics (n=67)	
Yes	4
No	63
Took antibiotics (n=67)	
Yes	3
No	64
Took anti-allergy medication (n=67)	
Yes	2
No	65
Took anti-inflammatory medication (n=67)	
Yes	2
No	65
Examination following symptoms	
Yes	22
No	65
Sought a health service	
Yes	78
No	9
Type of health service sought (n=78)	
Primary health care	31
Private care consultation	31
Hospital	8
Urgent care service	8
Medication prescribed at health service (n=78)	
Yes	75
No	3
Antibiotics prescribed (n=75)	
Yes	58
No	17
Analgesics/antipyretics prescribed (n=75)	
Yes	32
No	43
Anti-allergy medication prescribed (n=75)	
Yes	15
No	60
Anti-inflammatory medication prescribed (n=75)	
Yes	9
No	66

Among the 20 children who had contact with symptomatic classmates, 16 became ill. In addition, 56/87 cases reported that their classmates presented similar signs and symptoms. Furthermore, 57/87 cases did not present flu-like symptoms before pharyngotonsillitis and scarlet fever (Table 2).

Most students did not wear masks after the onset of symptoms (53/87) and 33/87 of them continued to attend school even though they were symptomatic. Most of the cases were medicated by their guardians, without a prescription, before seeking health care (67/87). Among the medications administered, analgesics/antipyretics (62/67) were the most cited (Table 2).

Most students did not undergo examination (65/87) after the onset of symptoms. Among the total of 87 cases, 78 sought care at health services, and 9 did not. Most sought private practices (31/78) and primary health care centers (31/78). Regarding the medications prescribed during these appointments, 75/78 students received a prescription for some kind of medication, and in 58/75 cases, antibiotics were the most recommended medications. Although 78 students reported receiving care, not all received a prescription for medications. Among those who did receive care, the total number of medications was higher, since some students were prescribed more than one class of medication, such as antibiotics and antipyretics (Table 2).

Individuals sought health care within a median of one day (minimum: 0 days; maximum: 30 days) after the onset of signs and symptoms. Among the 78 cases treated in the health system, tonsillitis (41), viral infections (6) and allergies (5) were the most frequently reported medical diagnoses by family members. Also noteworthy were those whose family members reported not having received a diagnosis (16/78) and the fact that none of the cases whose family members reported the presence of scarlatiniform rash received diagnosis of scarlet fever.

## Discussion

The deaths occurred due to septic shock, without laboratory confirmation of the causative agent, and were therefore classified as suspected invasive streptococcal disease. Identification of *Streptococcus sp*. among the individuals discharged from hospital is insufficient to confirm the occurrence of cases of invasive disease among the hospitalized children, but it may have been responsible for the probable outbreak of scarlet fever at the school.

Although there was no clinical validation of scarlet fever cases, presence of scarlatiniform rash was confirmed by photograph/picture, and the lack of medical diagnosis among the interviewees may indicate difficulty on the part of the healthcare team in diagnosing the disease. This may have occurred due to the absence of some of the specific signs and symptoms of scarlet fever when individuals sought health care. Epidemiological investigation played a fundamental role in identifying the diseases, possible sources of infection, transmission patterns and risk groups.

The age group affected is consistent with the literature, since the clinical manifestations of the diseases in question are found in school-age individuals ^1^^,^^3^^,^^5^^,^^18^^),^ drawing attention to the need for prevention and control measures to be reinforced in these environments. Presence of cases was observed in almost all the school’s grades and shifts, which coincided with the arrival of spring, a period in which pharyngotonsillitis and scarlet fever incidence tends to be higher ^1^, and the period in which the school underwent renovations to its physical structure and adapted containers for use as classrooms. Adapted containers being used as classrooms is a factor that may have contributed to the spread of diseases in the school in the following months, since small and poorly ventilated environments favor their spread, both of which are transmitted person-to-person via respiratory secretions ^1^^,^^5^.

Some of the cases reported contact with children who were hospitalized and with people with similar signs and symptoms prior to becoming ill. Students in the same class presented the same symptoms. Most students did not wear masks after the onset of symptoms, and many did not use their own glass/bottle to drink water at school, but rather shared these objects or drank directly from the water fountain. These findings reinforced the transmission of diseases, with characteristics of propagated transmission, which may have occurred at school, since many symptomatic students continued to attend school without adequate respiratory protection and prevention measures, and this is propitious for the spread of these diseases ^1^^,^^3^^,^^18^.

Among the most frequent symptoms presented by the students, occurrence of throat plaques stood out. Although tonsillar exudate is not a pathognomonic finding of bacterial infection ^19^ and the clinical-epidemiological cases of pharyngotonsillitis were not investigated with laboratory tests, its temporal and spatial correlation with the occurrence of clinical-epidemiological cases of scarlet fever at the school and with suspected deaths from invasive streptococcal disease in hospitalized children suggests that these cases would be correlated to the same infectious agent ^19^.

The epidemiological context, the progression of severe cases, as well as practices favorable to the spread of infection, support the hypothesis that there was an epidemiological link between the cases of children hospitalized with sepsis and the clinical-epidemiological cases of pharyngotonsillitis and scarlet fever among students. This suggests the occurrence of a probable outbreak of scarlet fever between September and October 2023 in a school in São João del-Rei, Minas Gerais.

Although pharyngotonsillitis is not subject to mandatory reporting, scarlet fever outbreaks must be reported. Such reporting allows health authorities to identify the extent and severity of the disease and implement appropriate control and prevention measures ^1^^,^^5^^,^^6^. These measures include timely diagnosis and treatment, staying away from school during the onset of symptoms, personal hygiene care, and hydration of the mucous membranes ^1^^,^^5^^,^^6^.

The results of this study may have been affected by selection and recall bias. The former because the starting point for the investigation at the school was the list of students with a history of school absenteeism, which did not include the two students who required hospitalization. The latter because there was a long time between the onset of symptoms and the date on which the interviews were conducted; however, the interviewees were reminded about the period to which the questions referred. 

Standing out among the limitations of this study is the lack of timely collection of samples from hospitalized cases, which corroborates the negative blood culture results, and the lack of laboratory confirmation of the etiological agent in severe cases, which made it difficult to link it to death. Although the interviews were conducted in the presence of the students’ legal guardians, who had monitored their health condition, lack of clinical validation of the pharyngotonsillitis and scarlet fever cases may have contributed to classification bias, in which some cases may not have been correctly diagnosed, leading to possible errors in categorization. It is noteworthy that, although the data suggest the occurrence of a probable scarlet fever outbreak, the cases were not clinically and laboratory validated.

In view of the results found, recommendations were made to issue a risk statement about scarlet fever and invasive diseases in the municipality, with the aim of raising awareness among the population and professionals in the investigation of differential diagnoses. Recommendations were made to promote awareness and public health education campaigns for the population and educational institutions about possible circulating etiological agents, infection prevention measures, such as: vaccination, personal hygiene measures, removal of symptomatic students from school, treatment, keeping classrooms well ventilated and individual use of glasses and bottles for drinking water. The effectiveness of these actions contributes to reducing the incidence of diseases and minimizes their impact on public health

## Data Availability

Data can be obtained by request to the corresponding author.
