# Supplementary material for: Investigation of deaths from ill-defined causes and probable scarlet fever outbreak among schoolchildren, São João del-Rei, 2023
Source: Epidemiol Serv Saude. 2025 Oct 16;35:e20240407. doi: 10.1590/S2237-96222026v35e20240407.en (PMC13690422; doi:10.1590/S2237-96222026v35e20240407.en)
Supplement: Translation

# Translation: Investigação de óbitos por causa mal definida e surto provável de escarlatina em escolares, São João del-Rei, 2023

**Authors:** Mariana Sanches de Mello, Andressa Rocha Olah, Katiucia Carolina Canaan, Karina Cardoso, Eva Lidia Arcoverde Medeiros, Daniela Caldas Teixeira, Flávia Ribeiro Soares Cruzeiro, Iara Fabiola Batista Rocha, Thais Cristina Nazaré Freitas, Maria Isabella Claudino Haslett, Samara Carolina Rodrigues

**Affiliations:** 1 Ministério da Saúde, Secretaria de Vigilância em Saúde e Ambiente, Brasília, DF, Brasil Ministério da Saúde Secretaria de Vigilância em Saúde e Ambiente Brasília DF Brasil; 2 Secretaria Municipal de Saúde de São João del-Rei, Vigilância Epidemiológica, São João del-Rei, MG, Brasil Secretaria Municipal de Saúde de São João del-Rei Vigilância Epidemiológica São João del-Rei MG Brasil; 3 Secretaria de Estado de Saúde de Minas Gerais, Centro de Informações Estratégicas em Vigilância em Saúde, Belo Horizonte, MG, Brasil Secretaria de Estado de Saúde de Minas Gerais Centro de Informações Estratégicas em Vigilância em Saúde Belo Horizonte MG Brasil

**Keywords:** Epidemiologia, Escarlatina, Estudo Observacional, Estudantes, Doenças do Trato Respiratório

## Abstract

**Objetivo:**

: Investigar óbitos por causas mal definidas e surto provável de escarlatina em escolares de São João del-Rei entre setembro e outubro de 2023.

**Métodos:**

: Estudo descritivo das crianças hospitalizadas com quadro séptico e série de casos de escolares com faringoamigdalite e escarlatina. Utilizaram-se dados de prontuários e entrevistas para calcular frequências absolutas e medidas de tendência central.

**Resultados:**

: Seis crianças hígidas (1-10 anos, brancas/pardas) foram hospitalizadas com quadro séptico, apresentando febre, petéquias, hematomas e odinofagia. Identificou-se Streptococcus sp. em swab orofaríngeo em três das seis crianças, mas as hemoculturas foram negativas. Três crianças faleceram, três receberam alta e duas frequentavam a mesma escola. Entre os escolares, 873 registros de absenteísmo foram identificados; destes, 212 atenderam à definição de faringoamigdalite e 87 foram entrevistados. Dos 87, 7 também atenderam aos critérios para escarlatina, 47 eram do sexo feminino e 78 tinham idade de 5-13 anos. Os sintomas incluíram mal-estar (62/87), placas na garganta (49/87) e manchas no corpo (26/87). Para tratamento, prescreveram-se antibióticos (58/75) e analgésicos/antitérmicos (32/75). Durante reforma na escola, salas de aula foram adaptadas para contêineres pequenos e pouco ventilados. Doze alunos não usavam copos individuais e 53/87 não utilizaram máscaras quando sintomáticos.

**Conclusões:**

: Os óbitos suspeitos de doença estreptocócica invasiva foram causados por choque séptico, sem confirmação do agente etiológico. O Streptococcus sp. pode ter contribuído para o surto provável de escarlatina. Recomendou-se alertar população e profissionais sobre práticas de higiene e afastamento escolar, além de capacitar equipes de saúde para diagnóstico e manejo de doenças reemergentes.

## Introdução

A faringoamigdalite infecciosa é uma das causas mais comuns de febre em crianças e contribui para o absenteísmo escolar, as infecções de repetição e o aumento do risco de complicações ^1^. Acomete frequentemente crianças após os 5 anos de idade e é mais comum no inverno e na primavera ^1^^-^^2^.

Trinta por cento dos casos têm etiologia bacteriana, sendo o *Streptococcus pyogenes* o mais prevalente ^2^^,^^3^^,^^4^^,^^5^^,^^6^^,^^7^. Cerca de 10,0% dos infectados são sensíveis às toxinas liberadas, e algumas cepas virulentas carregam genes que codificam superantígenos, o que inclui exotoxinas pirogênicas, o principal fator associado à erupção típica da escarlatina ^2^^,^^3^^,^^4^^,^^5^^,^^6^^,^^7^. Esse microrganismo também pode causar impetigo, febre reumática, glomerulonefrite e doenças invasivas como o choque tóxico ^2^^,^^3^.

A escarlatina caracteriza-se por febre alta, dor de garganta e *rash* escarlatiniforme - eritema difuso com micropápulas de 1 a 2 mm, o que confere à pele a textura semelhante à de uma lixa. Os indivíduos geralmente apresentam bochechas coradas e palidez ao redor da boca, além de erupção mais acentuada em locais de flexura, conhecido como sinal de Pastia. As papilas da língua podem apresentar hipertrofia, o que remete ao aspecto de “língua em framboesa” ^3^^,^^8^. A incubação varia de um a dez dias, e a transmissão ocorre desde os primeiros sintomas até 21 dias ou 24h após o início da antibioticoterapia ^5^^,^^6^. A escarlatina é transmitida pelo contato direto com secreções respiratórias, através de tosse e espirro, e pelo compartilhamento de objetos como copos, garrafas de água e talheres entre pessoas doentes e/ou assintomáticas ^1^^,^^2^^,^^3^^,^^4^^,^^5^^,^^6^^,^^9^.

O diagnóstico é clínico, sem a obrigatoriedade de exames para o início do tratamento. Apesar disso, exames laboratoriais em casos sintomáticos auxiliam na identificação do patógeno ^1^^,^^5^^,^^6^^,^^7^. A detecção pode ser realizada por testes bioquímicos, culturas microbiológicas e testes rápidos, que devem ser coletados preferencialmente antes da administração de antibióticos ^1^^,^^4^^,^^5^^,^^6^^,^^7^. O tratamento fundamenta-se em medidas de suporte, como o repouso, a hidratação e a administração de analgésicos, antitérmicos e antibióticos ^1^^,^^5^^,^^6^^,^^7^.

A escarlatina é sazonal e acomete crianças em idade escolar. Essa doença praticamente desapareceu no final do século XX ^3^, contudo surtos recentes ocorreram na Ásia e no Reino Unido e, concomitante a eles, observou-se o aumento nos casos de infecções invasivas ^3^^,^^4^^,^^5^.

Em 2022, casos de doença invasiva causada pelos estreptococos do grupo A e escarlatina foram reportados em países da Europa, nos Estados Unidos, no Uruguai e na Argentina, sendo identificados desfechos com evolução a óbito, relacionados aos casos de apresentação invasiva ^10^^,^^11^. No Brasil, surtos de escarlatina em crianças foram notificados em Minas Gerais ^12^ e São Paulo ^13^ em 2023, o que chamou a atenção para a reemergência da doença no território brasileiro.

Em São João del-Rei, Minas Gerais, a constatação de três óbitos e três hospitalizações de crianças, acompanhados de rumores de doença invasiva pelo *Streptococcus pyogenes,* motivou a solicitação de apoio do Programa de Treinamento em Epidemiologia Aplicada aos Serviços do Sistema Único de Saúde (EpiSUS) para a investigação epidemiológica. Este estudo objetivou investigar os óbitos com causas mal definidas, encontrar os vínculos epidemiológicos e investigar o provável surto de escarlatina entre os casos de faringoamigdalite em uma escola de São João del-Rei.

## Métodos

Delineamento

Tratou-se de estudo descritivo de crianças hospitalizadas com quadro séptico e série de casos, realizados entre 1º de setembro e 31 de outubro de 2023 em São João del-Rei, Minas Gerais. A escola onde foi realizada a série de casos estava localizada no mesmo município e atendia mais de 900 alunos da educação básica (infantil, fundamental e médio) e do ensino técnico.

Contexto

O Brasil é o maior país da América do Sul, com área territorial de 8 milhões de km^2^ e com população de 203.080.756 habitantes. O território divide-se em 27 Unidades Federativas, sendo 26 estados e o Distrito Federal. Em menor proporção, o país se subdivide em 5.588 municípios. Um deles é São João del-Rei, localizado no sudeste de Minas Gerais ^14^.

São João del-Rei é um município histórico, com população de 90.897 habitantes e um dos principais destinos turísticos do interior mineiro ^14^. Pertence à macrorregião de saúde Centro-Sul e, para a assistência à saúde da população, conta com 12 unidades básicas de saúde e 23 equipes de estratégia de saúde da família, com a cobertura da atenção primária à saúde de aproximadamente 73,0%. Também possui uma unidade de pronto atendimento, uma policlínica pública e duas unidades hospitalares.

Participantes

Este estudo incluiu as crianças hospitalizadas em São João del-Rei com quadro séptico e os alunos da escola onde duas dessas crianças estudavam e que tiveram registro de absenteísmo com relato de febre e odinofagia. Foram adotadas definições de caso de doença estreptocócica invasiva ^8^^,^^9^^,^^10^, óbito suspeito e confirmado de doença estreptocócica invasiva, caso de faringoamigdalite, caso de escarlatina e surto de escarlatina ^13^ adaptadas.

Caso de doença estreptocócica invasiva: indivíduo hospitalizado com quadro séptico e isolamento de *Streptococcus pyogenes* em cultura (hemocultura, líquor ou tecidos profundos), entre setembro e outubro de 2023.

Óbito suspeito de doença estreptocócica invasiva: indivíduo hospitalizado que evoluiu a óbito antes do diagnóstico de doença invasiva ou sem dados suficientes para classificar como provável ou confirmado, entre setembro e outubro de 2023.

Óbito confirmado de doença estreptocócica invasiva: indivíduo hospitalizado que evoluiu a óbito com diagnóstico médico de doença invasiva e que teve o isolamento de *Streptococcus pyogenes* em cultura (hemocultura, líquor ou tecidos profundos), entre setembro e outubro de 2023.

Caso de faringoamigdalite: indivíduo que apresentou febre e odinofagia, com registro de absenteísmo escolar entre setembro e outubro de 2023. 

Caso de escarlatina: indivíduo que apresentou febre, odinofagia e *rash* escarlatiniforme, com registro de absenteísmo escolar entre setembro e outubro de 2023.

Surto de escarlatina: ocorrência de dois ou mais casos de escarlatina em determinado espaço geográfico, no período de 21 dias após o início dos sinais e sintomas do primeiro caso identificado.

Não houve validação clínica dos casos, e consideraram-se os critérios clínico-epidemiológicos autorreferidos pelos participantes. Também foram consideradas as definições de validação clínica, critério clínico-epidemiológico, critério clínico-laboratorial, surto provável de escarlatina e surto confirmado de escarlatina (15) adaptadas ao contexto da investigação. 

Validação clínica: diagnóstico médico de faringoamigdalite/escarlatina, confirmado em prontuário.

Critério clínico-epidemiológico: caso que atendeu à definição de faringoamigdalite e/ou escarlatina e afirmou contato com as crianças hospitalizadas e/ou que adoeceram na escola.

Critério clínico-laboratorial: indivíduo que atendeu à definição de faringoamigdalite e/ou escarlatina e teve o isolamento de *Streptococcus pyogenes* em cultura (*swab* orofaríngeo, hemocultura ou tecidos profundos).

Surto provável de escarlatina: conjunto de casos de escarlatina que atendeu à definição de surto de escarlatina e apresentou critério clínico-epidemiológico, porém sem confirmação laboratorial.

Surto confirmado de escarlatina: conjunto de casos de escarlatina que atendeu à definição de surto de escarlatina e pelo menos um caso que atendeu ao critério clínico-laboratorial e os demais atenderam ao critério clínico-epidemiológico.

Fonte e análise de dados

Foram coletados dados secundários de prontuários e primários por entrevistas. Para a investigação das crianças hospitalizadas e para a investigação conduzida na escola, utilizaram-se instrumentos semiestruturados contendo variáveis sociodemográficas e clínico-epidemiológicas. Para a coleta de dados nos prontuários, o instrumento foi dividido em variáveis sociodemográficas (idade, sexo, município de residência e escola que frequentava) e variáveis clínico-epidemiológicas (principais sinais e sintomas, hipótese diagnóstica, exames realizados e desfecho clínico).

O questionário utilizado para as entrevistas dos escolares foi elaborado no EpiInfo versão 7.0 e dividido em blocos: dados do respondente, dados do escolar (data de nascimento, raça/cor da pele, sexo, município de residência), dados clínico-epidemiológicos (turno e série de estudo, copo individualizado para beber água na escola, sinais e sintomas e data de início, exames realizados, correlaciona o início dos sintomas a evento/situação anterior, uso de máscara durante a manifestação dos sintomas, contato com pessoas com sintomas semelhantes, familiar com sintomas semelhantes, criança da mesma sala com sintomas semelhantes, contato com as crianças internadas, viagem anterior aos sintomas, quadro gripal do escolar antes dos sintomas) e busca por atendimento (medicação sem prescrição médica, número de vezes que procurou atendimento, data e local em que buscou atendimento, diagnóstico recebido no atendimento, medicação prescrita, internação). Quando mencionada pelo responsável e/ou pelo estudante a presença de manchas na pele, era solicitado pelos entrevistadores imagens fotográficas que comprovassem o relato e/ou apresentadas imagens que pudessem indicar o tipo de mancha apresentada pelo indivíduo.

A equipe de coleta de dados foi previamente treinada e composta por membros da Secretaria de Estado de Saúde de Minas Gerais, da Secretaria Municipal de Saúde de São João del-Rei e do EpiSUS. Foram consideradas perdas os estudantes cujos responsáveis foram contactados por três vezes em dias e horários diferentes, presencialmente e/ou por telefone e não se obteve sucesso, aqueles cujos endereços ou contatos telefônicos dos responsáveis não foram encontrados e aqueles que se recusaram a participar. 

A participação dos indivíduos ocorreu de forma voluntária, mediante a autorização verbal do responsável legal, sem benefícios financeiros ou coerção ^16^. Houve dispensa do registro de consentimento livre e esclarecido por se tratar de ação de vigilância em saúde pública. Os dados obtidos ^17^ foram analisados pelo cálculo das medidas de frequência absoluta e tendência central, utilizando o software SPSS versão 22.0.

## Resultados

No período do estudo, seis crianças previamente hígidas foram hospitalizadas em São João del-Rei com quadro séptico. No que tange a caracterização dos indivíduos, todos eram da raça/cor da pele branca ou parda, com idade entre 1-10 anos, apresentaram febre e manchas no corpo (petéquias, hematomas e sufusões hemorrágicas). Quatro delas apresentaram odinofagia. Identificou-se o *Streptococcus sp*. em cultura de *swab* orofaríngeo em três crianças, porém com hemoculturas negativas. Três faleceram, três receberam alta, e duas frequentavam a mesma escola (Figura 1). Levantou-se a hipótese de que houve um surto de doença infecciosa bacteriana na escola onde duas das crianças hospitalizadas estudavam.

Conduziu-se investigação epidemiológica nessa escola e foram encontrados 873 registros de absenteísmo no período. Conforme a definição de caso de faringoamigdalite, 212 (24,3%) estudantes foram elegíveis para o estudo, entretanto, em 113/212 casos, não se obteve sucesso nos contatos realizados, 8/212 se recusaram a participar e 4/212 não tinham informações sobre o endereço ou contato telefônico. Do total de estudantes elegíveis para o estudo (212), 87 foram entrevistados.

A faixa etária predominante entre os casos foi 5-13 anos (78/87), entretanto ocorreram casos em indivíduos de 14-19 anos (9/87) (Tabela 1). Todos os entrevistados atenderam à definição de caso clínico-epidemiológico para faringoamigdalite, além disso, 7/87 também atenderam à definição de caso clínico-epidemiológico de escarlatina. Do total de casos, houve maior ocorrência entre pessoas do sexo feminino (47/87), com predomínio da raça/cor da pele branca (60/87) e que residia em São João del-Rei (65/87) (Tabela 1).

**Tabela 1:** Características sociodemográficas dos casos de faringoamigdalite e escarlatina. São João del-Rei, setembro a outubro de 2023 (n=87)

Variável	n
Sexo	
Feminino	47
Masculino	40
Raça/cor da pele	
Branca	60
Parda	24
Amarela	2
Preta	1
Faixa etária (anos)	
5-7	28
8-10	27
11-13	23
14-16	6
17-19	3
Munícipio de residência	
São João del-Rei	65
Santa Cruz de Minas	19
Tiradentes	1
Coronel Xavier Chaves	1
Sem informação	1

Em agosto de 2023, iniciou-se uma reforma na estrutura física da escola e, a partir do dia 6 desse mesmo mês, 20 das 26 turmas foram realocadas em contêineres e seis permaneceram em salas convencionais. As turmas eram distribuídas entre os turnos matutino (A) e vespertino (B). As duas turmas da educação infantil (A e B) e as 18 do ensino fundamental (1º ao 9º ano) de ambos os turnos passaram a assistir às aulas em contêineres adaptados. As seis turmas do ensino médio (1º ao 3º ano) de ambos os turnos permaneceram em sala de aula convencionais. Apesar de os contêineres possuírem portas e janelas, os espaços eram reduzidos e pouco ventilados. 

Das 26 turmas/séries da escola, 22 tiveram registro de, pelo menos, um caso clínico-epidemiológico de faringoamigdalite e/ou escarlatina. Entre as 20 turmas alocadas nos contêineres, 82/87 estudantes apresentaram sintomas e, entre aqueles que permaneceram em salas convencionais, 5/87 foram sintomáticos. Entre as turmas e os turnos que apresentaram o maior número de casos no período, destacou-se o 4º ano B do ensino fundamental, uma das turmas que tiveram as salas de aula adaptadas e a qual duas das crianças que foram hospitalizadas frequentavam (Figura 2). 

**Figura 2:** Turma e turno de estudo, manhã (A) e tarde (B), dos casos de faringoamigdalite e escarlatina. São João del-Rei, setembro a outubro de 2023 (n=87)

Os 87 casos clínico-epidemiológicos de faringoamigdalite e escarlatina distribuíram-se temporalmente em agosto (1/87), setembro (38/87) e outubro (47/87); um (1/87) dos casos não se soube precisar a data de início de sinais e sintomas. Evidenciou-se que, a partir da segunda quinzena de setembro, houve aumento do número de casos, quando comparado a agosto (Figura 3).

Além dos sinais e sintomas estabelecidos nas definições de caso (febre e odinofagia), mais da metade dos 87 casos clínico-epidemiológicos de faringoamigdalite e escarlatina cursaram com mal-estar (62/87), prostração (59/87), cefaleia (52/87) e placas na garganta (49/87). As manchas no corpo estiveram presentes em 26/87 casos e as mais informadas foram sufusões hemorrágicas (1/26), petéquias (16/26), *rash* escarlatiniforme (7/26) e vesículas (1/26).

**Figura 3:** Casos de faringoamigdalite e escarlatina, segundo a data de início dos sintomas. São João del-Rei, setembro a outubro de 2023 (n=86)

Ao questionar sobre o uso de itens pessoais no ambiente escolar, observou-se que 12/87 estudantes não utilizavam copo ou garrafa de água próprios, utilizando diretamente o bebedouro ou compartilhando os objetos entre os colegas. O início da sintomatologia de 43/87 casos estava relacionado a algum evento/situação anterior, com destaque para a adaptação das salas de aula em contêineres (14/43) e a participação em uma festa de aniversário. (Tabela 2).

Entre os entrevistados, oito crianças viajaram antes dos sinais e sintomas apresentados, e os principais destinos foram cidades dos estados de Minas Gerais, São Paulo, Rio de Janeiro e Goiás. Entretanto, não houve relato de contato com pessoas sintomáticas nessa ocasião. Foi informado que 25/87 crianças tiveram contato com pessoas que apresentaram sintomatologia semelhante antes de desenvolverem o quadro de faringoamigdalite ou escarlatina (Tabela 2).

Entre as 20 crianças que tiveram contato com colegas sintomáticos, 16 adoeceram. Além disso, 56/87 casos informaram que os colegas de turma apresentaram sinais e sintomas semelhantes. Ademais, 57/87 casos não apresentaram síndrome gripal antes da faringoamigdalite e da escarlatina. (Tabela 2).

A maioria dos estudantes não utilizou máscaras após o início dos sintomas (53/87) e 33/87 deles continuaram a frequentar a escola mesmo sintomáticos. Grande parte dos casos foi medicada pelos responsáveis, sem prescrição médica, antes de procurar atendimento de saúde (67/87). Entre os medicamentos administrados, os analgésicos/antitérmicos (62/67) foram os mais citados (Tabela 2).

A maior parte dos estudantes não realizou exames (65/87) após o início dos sintomas. Entre os 87 casos totais, 78 procuraram atendimento nos serviços de saúde, e 9 não o fizeram. A maioria buscou os consultórios particulares (31/78) e as unidades de atenção primária à saúde (31/78). Sobre as medicações prescritas nesses atendimentos, 75/78 estudantes receberam a prescrição de algum medicamento, e, em 58/75 casos, os antibióticos foram as medicações mais recomendadas. Embora 78 estudantes tenham relatado atendimento, nem todos receberam a prescrição de medicamentos. Entre aqueles que receberam, o total de medicações é maior, pois, para alguns estudantes, foram prescritas mais de uma classe de medicamentos, a exemplo de antibiótico e antitérmico (Tabela 2). 

**Tabela 2:** Variáveis clínico-epidemiológicas dos casos de faringoamigdalite e escarlatina. São João del-Rei, setembro a outubro de 2023 (n=87)

Variáveis	n
Uso de copo/garrafa próprio	
Sim	75
Não	12
Início dos sintomas correlacionado a evento/situação anterior	
Sim	43
Não	44
Viagem 20 dias antes dos sintomas	
Sim	8
Não	79
Contato anterior com pessoa sintomática	
Sim	25
Não	56
Sem informação	6
Contato com crianças internadas	
Sim	20
Não	60
Sem informação	7
Aparecimento dos sintomas (n=20)	
Antes	4
Após	16
Colega de sala sintomático	
Sim	56
Não	10
Sem informação	21
Estudante com síndrome gripal prévia	
Sim	30
Não	57
Uso de máscara após sintomas	
Sim	34
Não	53
Frequentou escola sintomático	
Sim	33
Não	54
Medicação prévia à assistência sem prescrição	
Sim	67
Não	20
Utilizou analgésicos/antitérmicos (n=67)	
Sim	62
Não	5
Utilizou antieméticos (n=67)	
Sim	4
Não	63
Utilizou antibióticos (n=67)	
Sim	3
Não	64
Utilizou antialérgicos (n=67)	
Sim	2
Não	65
Utilizou anti-inflamatórios (n=67)	
Sim	2
Não	65
Exames após sintomas	
Sim	22
Não	65
Buscou serviço de saúde	
Sim	78
Não	9
Serviço de saúde procurado (n=78)	
Atenção primária à saúde	31
Consulta particular	31
Hospital	8
Unidade de pronto atendimento	8
Medicação prescrita no serviço de saúde (n=78)	
Sim	75
Não	3
Prescrito antibióticos (n=75)	
Sim	58
Não	17
Prescrito analgésicos/antitérmicos (n=75)	
Sim	32
Não	43
Prescrito antialérgicos (n=75)	
Sim	15
Não	60
Prescrito anti-inflamatórios (n=75)	
Sim	9
Não	66

Indivíduos buscaram atendimento com mediana de um dia (mínimo: 0 dias; máximo: 30 dias) após o aparecimento dos sinais e sintomas. Entre os 78 casos que foram atendidos no sistema de saúde, a amigdalite (41), as infecções virais (6) e as alergias (5) foram os diagnósticos médicos mais referidos pelos familiares. Destacaram-se ainda aqueles cujos familiares informaram não ter recebido um diagnóstico (16/78) e o fato de que nenhum dos casos, cujos familiares referiram a presença do *rash* escarlatiniforme, ter recebido o diagnóstico de escarlatina.

## Discussão

Os óbitos ocorreram por choque séptico, sem a confirmação laboratorial do agente causador, portanto, classificados como suspeitos de doença estreptocócica invasiva. A identificação do *Streptococcus sp.* entre os indivíduos que receberam alta é insuficiente para confirmar a ocorrência de casos de doença invasiva entre as crianças hospitalizadas, porém pode ter sido responsável pelo surto provável de escarlatina na escola. 

Apesar de não ter ocorrido a validação clínica dos casos de escarlatina, a presença do *rash* escarlatiniforme foi confirmada por fotografia/figura, e a ausência de diagnóstico médico entre os entrevistados pode indicar dificuldade da equipe assistencial em diagnosticar a doença. Isso pode ter ocorrido devido à ausência de alguns dos sinais e sintomas específicos da escarlatina à busca pelo atendimento de saúde pelos indivíduos. A investigação epidemiológica teve papel fundamental para identificar as doenças, as possíveis fontes de infecção, os padrões de transmissão e os grupos de risco.

A faixa etária acometida está em consonância com a literatura, pois as manifestações clínicas das doenças em questão são encontradas em indivíduos em idade escolar ^1^^,^^3^^,^^5^^,^^18^, chamando a atenção para que as medidas de prevenção e controle sejam reforçadas nesses ambientes. Observou-se a presença de casos em quase todas as séries e turnos da escola, o que coincidiu com a chegada da primavera, período no qual a incidência da faringoamigdalite e escarlatina costuma ser maior ^1^, com o período em que a escola passou por uma reforma em sua estrutura física e adaptou contêineres em salas de aulas. A adaptação das salas de aulas em contêineres é um fator que pode ter contribuído para a disseminação das doenças na escola nos meses subsequentes, visto que ambientes pequenos e pouco ventilados favorecem a disseminação das doenças, ambas de transmissão pessoa a pessoa via secreções respiratórias ^1^^,^^5^.

Alguns dos casos informaram contato com as crianças que foram hospitalizadas e com pessoas com sinais e sintomas semelhantes, anteriormente ao adoecimento. Alunos da mesma turma apresentaram os mesmos sintomas. A maioria dos estudantes não utilizou máscaras após o início dos sintomas, e muitos não utilizavam o próprio copo/garrafa para beber água na escola, compartilhando os objetos ou bebendo diretamente no bebedouro. Estes achados reforçaram a forma de transmissão das doenças, com característica de transmissão propagada, que pode ter ocorrido na escola, pois muitos alunos sintomáticos continuaram a frequentar o local sem as medidas adequadas de proteção respiratória e prevenção, as quais propiciam a disseminação das doenças ^1^^,^^3^^,^^18^.

Entre os sintomas mais frequentes apresentados pelos estudantes salientou-se a ocorrência de placas na garganta. Apesar de o exsudato amigdaliano não ser um achado patognomônico de infecção bacteriana ^19^ e os casos clínico-epidemiológicos de faringoamigdalite não terem sido investigados com exames laboratoriais, sua correlação temporal e espacial com a ocorrência de casos clínico-epidemiológicos de escarlatina na escola e com óbitos suspeitos de doença estreptocócica invasiva das crianças hospitalizadas sugere que esses casos estariam correlacionados ao mesmo agente infeccioso ^19^. 

O contexto epidemiológico, a evolução dos casos graves e as práticas favoráveis à disseminação infecciosa sustentam a hipótese de que existia vínculo epidemiológico entre os casos das crianças hospitalizadas com sepse e os casos clínico-epidemiológicos de faringoamigdalite e escarlatina entre os estudantes. Isso sugere a ocorrência de provável surto de escarlatina entre setembro e outubro de 2023 em uma escola de São João del-Rei, Minas Gerais.

Apesar de a faringoamigdalite não ser de notificação compulsória, os surtos de escarlatina devem ser notificados. Tais comunicações permitem que as autoridades de saúde identifiquem a extensão e gravidade da doença e implementem as medidas de controle e prevenção adequadas ^1^^,^^5^^,^^6^. Essas medidas estão relacionadas ao diagnóstico e aos tratamentos oportunos, ao afastamento das atividades escolares durante a manifestação dos sintomas, aos cuidados de higiene pessoal e à hidratação das mucosas ^1^^,^^5^^,^^6^.

Os resultados deste estudo podem ter sido impactados pelo viés de seleção e de memória. O primeiro porque o ponto de partida para a investigação na escola foi a lista com a relação de alunos com histórico de absenteísmo escolar que não contemplava os dois alunos que necessitaram de internação. O segundo porque o tempo entre o início dos sintomas e a data em que as entrevistas foram realizadas foi longo, entretanto o período o qual se referiam às perguntas era lembrado aos entrevistados. 

Como limitação deste estudo, destacaram-se a ausência de coleta oportuna de amostras dos casos hospitalizados, o que corrobora os resultados das hemoculturas negativas, e a ausência de confirmação laboratorial do agente etiológico nos casos graves, o que dificultou sua vinculação ao óbito. Embora as entrevistas tenham sido realizadas na presença do responsável legal, que acompanhou o quadro de saúde do estudante, a ausência de validação clínica dos casos de faringoamigdalite e escarlatina pode ter contribuído para um viés de classificação, no qual alguns casos podem não ter sido corretamente diagnosticados, levando a possíveis erros na categorização. Destaca-se que, apesar de os dados sugerirem a ocorrência de provável surto de escarlatina, os casos não foram validados clínica e laboratorialmente.

Diante dos resultados encontrados, recomendou-se emitir comunicado de risco sobre escarlatina e doenças invasivas no município, com o intuito de sensibilizar a população e os profissionais na investigação de diagnósticos diferenciais. Indicou-se promover campanhas de sensibilização e educação em saúde pública para a população e instituições de ensino sobre os possíveis agentes etiológicos circulantes, medidas de prevenção de infecções, tais como: vacinação, medidas de higiene pessoal, afastamento dos alunos sintomáticos da escola, tratamentos, manutenção das salas de aulas bem ventiladas e uso individual de copos e garrafas para beber água. A eficácia dessas ações contribui para reduzir a incidência das doenças e minimiza seu impacto na saúde pública

## Data Availability

Os dados podem ser obtidos através de solicitação ao autor correspondente
